# Neuroprotective Effects of VGLUT1 Inhibition in HT22 Cells Overexpressing VGLUT1 Under Oxygen Glucose Deprivation Conditions

**DOI:** 10.1007/s12017-024-08803-3

**Published:** 2024-08-23

**Authors:** B. Pomierny, W. Krzyżanowska, A. Skórkowska, B. Budziszewska, J. Pera

**Affiliations:** 1 Department of Toxicological Biochemistry, Jagiellonian University Medical College, Kraków, Poland; 2 BioImaging Laboratory, Centre for the Development of Therapies for Civilizational and Age-Related Diseases (CDT-CARD), Kraków, Poland; 3 Department of Neurology, Jagiellonian University Medical College, Kraków, Poland

**Keywords:** Brain ischemia, Vesicular glutamate transporters, Glutamate, Oxygen glucose deprivation, Chicago Sky Blue 6B

## Abstract

Glutamate (Glu) is a major excitatory neurotransmitter in the brain, essential for synaptic plasticity, neuronal activity, and memory formation. However, its dysregulation leads to excitotoxicity, implicated in neurodegenerative diseases and brain ischemia. Vesicular glutamate transporters (VGLUTs) regulate Glu loading into synaptic vesicles, crucial for maintaining optimal extracellular Glu levels. This study investigates the neuroprotective effects of VGLUT1 inhibition in HT22 cells overexpressing VGLUT1 under oxygen glucose deprivation (OGD) conditions. HT22 cells, a hippocampal neuron model, were transduced with lentiviral vectors to overexpress VGLUT1. Cells were subjected to OGD, with pre-incubation of Chicago Sky Blue 6B (CSB6B), an unspecific VGLUT inhibitor. Cell viability, lactate dehydrogenase (LDH) release, mitochondrial membrane potential, and hypoxia-related protein markers (PARP1, AIF, NLRP3) were assessed. Results indicated that VGLUT1 overexpression increased vulnerability to OGD, evidenced by higher LDH release and reduced cell viability. CSB6B treatment improved cell viability and reduced LDH release in OGD conditions, particularly at 0.1 μM and 1.0 μM concentrations. Moreover, CSB6B preserved mitochondrial membrane potential and decreased levels of PARP1, AIF, and NLRP3 proteins, suggesting neuroprotective effects through mitigating excitotoxicity. This study demonstrates that VGLUT1 inhibition could be a promising therapeutic strategy for ischemic brain injury, warranting further investigation into selective VGLUT1 inhibitors.

## Introduction

Glutamate (Glu) is the predominant excitatory neurotransmitter in the brain, playing a pivotal role in synaptic plasticity, neuronal activity, neuronal development, and memory formation. Moreover, Glu is critically involved in excitotoxicity, a pathological condition characterized by excessive glutamatergic neurotransmission that leads to cellular damage, observed in neurodegenerative diseases and brain ischemia (Dong et al., 2009; Shen et al., 2022). The release and clearance of Glu are meticulously regulated by vesicular glutamate transporters (VGLUTs) and membrane, excitatory amino acid transporters (EAATs), respectively. These transporters constitute a sophisticated regulatory network that is essential for maintaining optimal extracellular Glu levels, thereby facilitating neurotransmission and preventing the cytotoxic effects of Glu accumulation. In cerebral ischemia, this regulatory system becomes impaired, potentially leading to an increased rate of Glu uptake into synaptic vesicles, predominantly by VGLUT1 and VGLUT2. This results in heightened release into the synaptic cleft, Glu spillover to the extrasynaptic space, hyperactivation of N-methyl-D-aspartate receptors (NMDAR) and saturated activity of Glu uptake transporters, ultimately culminating in neuronal cell death (Krzyzanowska et al., 2014).

Previous attempts to mitigate excitotoxicity in cerebral ischemia by inhibiting the NMDAR or enhancing astrocytic uptake of Glu via EAATs failed in clinical trials. These strategies primarily targeted Glu that had already been released into the synaptic or extrasynaptic spaces, potentially overlooking critical upstream regulatory mechanisms that control Glu release and homeostasis (Choi, 2020; Pellegrini-Giampietro et al., 2004). Data on strategies to modulate the presynaptic mechanisms involved in Glu over-release during stroke are scarce. This gap highlights the need for research focused on presynaptic regulation, which could offer new therapeutic targets for preventing excitotoxicity. VGLUTs (SLC17 family) are known to be responsible for Glu loading into vesicles in presynaptic terminals. VGLUT1 and VGLUT2 are extensively expressed throughout the brain, mainly on glutamatergic neurons, but their distribution differs. These isoforms play a vital role in glutamatergic transmission (Fremeau et al., 2001; Kaneko & Fujiyama, 2002; Takamori et al., 2000). VGLUT1 or VGLUT2 knockout mice die just after weaning or after birth, respectively. The expression of VGLUT3 is limited to non-glutamatergic neurons in the dorsal and ventral striatum, cortex, and hippocampus. The expression levels of VGLUTs determine the amount of Glu loaded into presynaptic vesicles and its subsequent release into the synaptic cleft. Thus, inhibition of VGLUTs transporters in excitotoxic conditions is supposed to ameliorate Glu-dependent neural damage. In our previous studies using a rat model of focal cerebral ischemia we showed that stroke upregulates VGLUT1 and inhibition of this transporter using an azo-dye Chicago Sky Blue 6B (CSB6B) resulted in reduced Glu efflux, smaller infarct size and improved neurological condition. Treatment with CSB6B revealed also a profound anti-inflammatory effect, what may suggest a broad, multifactorial mode of action (Pomierny et al., 2023, 2024).

In this study we aimed to verify neuroprotective effects of VGLUT1 inhibition in HT22 cell culture with overexpression of VGLUT1 protein in a model of oxygen glucose deprivation (OGD). A set of tests was designed to verify in in vitro experiment an effect of VGLUT1 inhibition, we previously observed in vivo. Here, we assessed the effect of preincubation of cells with CSB6B on hypoxia-related damage. Next, we determined the effect of VGLUT1 suppression on cell viability, release of lactate dehydrogenase (LDH), but also on the mitochondrial membrane potential. Furthermore, we analyzed the effect of OGD and CSB6B on marker proteins of hypoxia-related cell damage, that is PARP1, AIF, NLRP3.

## Methods

### HT22 Cell Culture

Cells were cultured in Dulbecco's Modified Eagle Medium (DMEM), supplemented with 10% fetal bovine serum (FBS) and 1% penicillin–streptomycin to prevent microbial contamination. The culture was maintained in a humidified incubator at 37 °C with an atmosphere of 5% CO_2_. HT22 cells were passaged upon reaching an approximate confluence of 80–90%. For passaging, cells were detached using 0.25% trypsin–EDTA solution, neutralized in complete medium, and seeded at a density of 5,000 to 10,000 cells/cm^2^, depending on the experimental setup. Care was taken to use cells at low passage numbers to maintain their physiological relevance.

### Preparation of HT22 Cells Overexpressing VGLUT1 via rLV Infection for Gene Transfer Efficiency Analysis

**Fig. 1:**
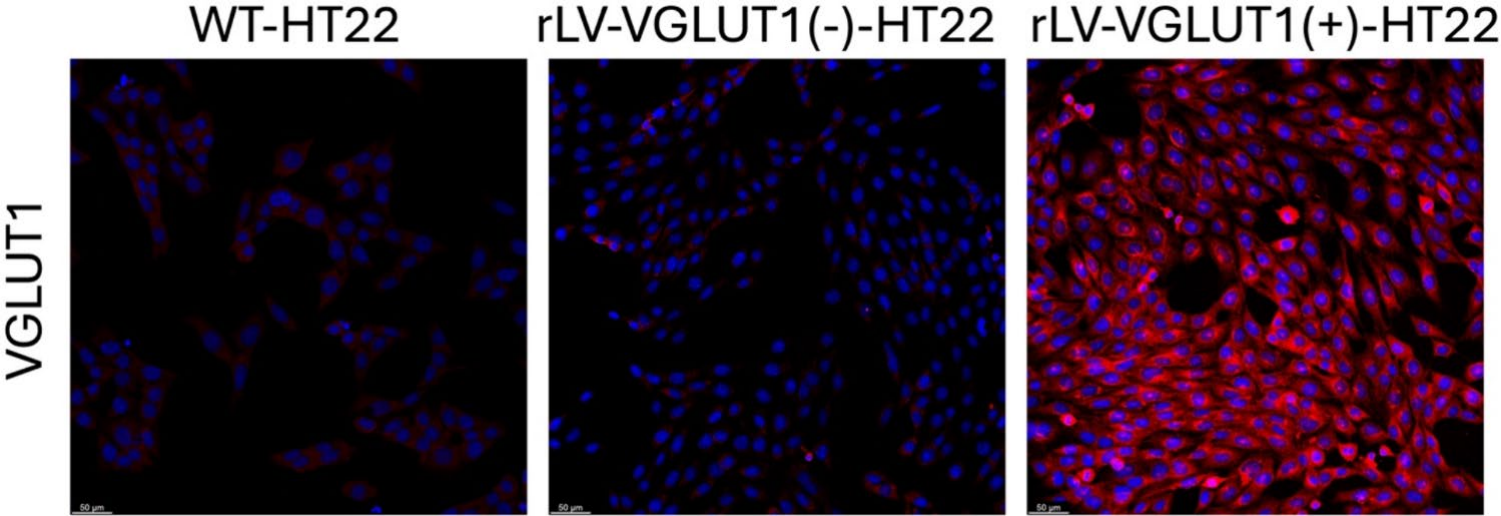
The immunofluorescent staining shows VGLUT1 expression in wild-type (WT) HT22 cell cultures (left), the same cell line transduced with a blank vector (middle), and HT22 cells transduced with an rLV vector containing the VGLUT1 expression construct (right). The scale bar = 50 μm

In order to determine the more efficient viral vector for gene transfert expression in target cells, GIGA Viral Vectors platform (University of Liège, Liège, Belgium) tested different ready-to use lentiviral vectors (MOI = 50) that should allow fluorescent protein expression under the control of different promoters (hPGK, SV40, mPGK, CBh, EF1a, CMV, SFFV CAG) and having different envelope (VSV-G, Measles, RD114 and GalV). SFFV promoter combine with VSV-g pseudotyped LV was the most efficient combination. Knowing that, gene transfer lentiviral plasmids were purchased from Vector Builder, pLV SFFV VGlu1-IRES-mCherry (VB220831-1419dpk) for and pLV SFFV luc2-IRES-mCherry (CMV Puro) as control (VB220901-1439asa). The plasmid allows expression of VGlu1 (or mSlc17a7[NM_182993.2]) and mCherry. Lentiviral vectors were generated by GIGA Viral Vectors platform (University of Liège, Liège, Belgium). Lenti-X 293 T cells (Clontech Laboratories, Inc., Mountain View, CA, USA) were co-transfected with gene transfer lentiviral plasmids, pSPAX2 (Addgene, Cambridge, MA, USA) and VSV-G encoding vectors (Cell Biolabs, Inc., San Diego, CA, USA). Lentiviral supernatants were collected at 96 h post-transfection, filtrated, concentrated with lenti-Pac Lentivirus Concentration Solution (BioCat #LT007-GC), titrated and used to transduce HT22 cells (MOI = 50) in order to allow the expression of VGlu1 and mCherry (or Luciferase as control). Transduced cells were selected with 1 µg/mL puromycin. The absence of RCL and mycoplasma in cell supernatant was confirmed with qPCR Lentivirus Titration kit (Lonza, Basel, Switzerland) and MycoAlert PLUS Mycoplasma Detection Kit (Lonza, Basel, Switzerland), respectively. The effectiveness of vectors used on the expression level of VGLUT1 protein has been verified using immunofluorescent staining and imaging with confocal microscopy as described below (Fig. 1).

### Oxygen Glucose Deprivation (OGD)

The experiment was conducted using HT22 immortalized hippocampal neurons cell lines transduced with rLV SFFV-Vglut1-IRES-mCherry to gain VGLUT1 overexpression or rLV SFFV-luc2-IRES-mCherry as a control construct. Cells were seeded into 96-well sterile test plates in standard culture medium (10% FBS in DMEM). Cellular suspension used for the experiment was 3000 cells/well. After 24 h of incubation, the medium was changed to FBS-depleted medium (1% FBS in DMEM). CSB6B solutions were added to selected wells at final concentrations: 0.1, 1.0 and 10.0 µM, 2 h prior to OGD. After this period of time medium was replaced with fresh glucose-free, FBS-depleted medium and CSB6B solutions were added at the concentrations described above, into respective wells. HT22 cells were then subjected to OGD in a hypoxic chamber in an incubator (5%CO2/95%N2) at 37 °C for 18 h. Cells were next transferred to normoxia conditions and medium was replaced with standard culture medium. After 3-h incubation period cells were subjected to viability and cytotoxicity tests as well as immunofluorescence staining and mitochondria membrane potential assay.

### Immunofluorescence Staining

Three hours after reoxygenation, culture medium was removed, and cells were washed with PBS. Cells were next fixed with freshly prepared 4% paraformaldehyde (PFA) aqueous solution for 15 min at room temperature (RT). Cells were rinsed with PBS and permeabilized with 0.3% Triton-X in PBS for 15 min at RT. Cells were washed again with PBS and incubated in 5% normal goat serum in PBS for 1 h at RT. Next, cells were incubated with specific primary antibodies solutions at 4 °C overnight. The following day cells were washed with PBS and incubated with specific secondary antibody solution for 1 h at RT in the dark (Table 1). After the last washing step in PBS, cells were counter-stained with DAPI mounting solution (Vector). Cells were visualized using Leica Stellaris 8 confocal microscope and Leica LAS X software. The fluorescence intensity was measured using Leica LAS X software. For preprocessing at each image (n = 7 per group) cells were segmented, histogram was set at the same level along the study, and the mean fluorescence was read across all analyzed images. Since the culture density was relatively low—there were no necessity to use any filters, as such the raw results are presented as the relative fluorescence units (RFU).

**Table 1:** Antibodies used in immunofluorescent study

Antibody	Manufacturer	Catalog number	Concentration
VGLUT1	Sigma Aldrich	AMAb91041	1:1000
PARP1	Invitrogen	436400	1:333
AIF	Invitrogen	MA5-15880	1:75
NLRP3	Invitrogen	MA5-32255	1:300
Goat anti-rabbit AlexaFluor 488	Invitrogen	A11034	1:400
Goat anti-mouse AlexaFluor Plus 488	Invitrogen	A32723	1:400

### Cell Viability

Viability of HT22 cells after the indicated treatments was evaluated using the resazurin-based PrestoBlue™ reagent (Invitrogen, ThermoFischer Scientific, USA). Briefly, the reagent was warmed to room temperature before use and then 10 µl of PrestoBlue solution was added to cells in 90 µl culture medium in each well. The plates were incubated for 30 min at 37 °C (5% CO_2_), protected from light. The absorbance of resorufin, a red compound formed in the reducing environment of living cells, was measured using an Infinite M200 Pro plate reader (Tecan, Switzerland) at 570 nm, with 630 nm as reference wavelength. The cell viability was corrected for background absorbance (wells with cell culture medium only) and expressed as a percentage relative to control.

### LDH Release Assay

The extracellular lactate dehydrogenase (LDH) was measured using a CyQUANT™ LDH Cytotoxicity Assay Kit (Invitrogen, ThermoFisher Scientific, USA), following the manufacturer’s protocol. Combined cell culture media aspirated after OGD and after reoxygenation time were incubated with the reagent mixture at RT for 30 min, protected from light. The level of a red formazan product, which is directly proportional to the amount of LDH released into the medium, was measured immediately after adding stop solution using an Infinite M200 Pro plate reader (Tecan, Switzerland) at 490 nm and 680 nm (background). Triton-X 100-treated cells were used as a positive control. The results were corrected for background absorbance (wells with cell culture medium only) and expressed as a percentage relative to control group.

### Measurement of Mitochondrial Membrane Potential

Mitochondrial membrane potential of cells was determined after the indicated treatments using cationic dye JC-1 (Cayman chemical, USA). After removing culture medium, cells were incubated with JC-1 staining solution for 30 min in the incubator, following the manufacturer’s protocol. Afterwards, the cells were washed and fluorescence was quantified by a Fluoroskan Ascent FL plate reader (Thermo Fisher Scientific, USA) as well as visualized using a Stellaris 8 WLL DLS confocal microscope (Leica, Germany). Red fluorescence (with excitation and emission at 535 nm and 595 nm wavelength, respectively) was proportional to the number of healthy cells, while green fluorescence (with excitation and emission at 485 nm and 535 nm, respectively) to cells with low mitochondrial membrane potential. The ratio of green to red fluorescence signal was calculated and the results were expressed as a ratio.

### Statistics

All data are expressed as the mean ± standard deviation (SD). All data were analyzed using one-way ANOVA. If statistical significance was found after ANOVA, Sidak’s post hoc test was conducted to test the comparisons between experimental groups. A calculated *p* value < 0.05 was considered statistically significant. Calculations were performed using GraphPad Prism version 10.2.1 for macOS, GraphPad Software, Boston, Massachusetts USA, www.graphpad.com.

## Results

### The Effect of VGLUT1 Inhibition on Cell Viability was Assessed in HT22 Cells Under OGD Conditions and with Various Concentrations of CSB6B

**Fig. 2:**
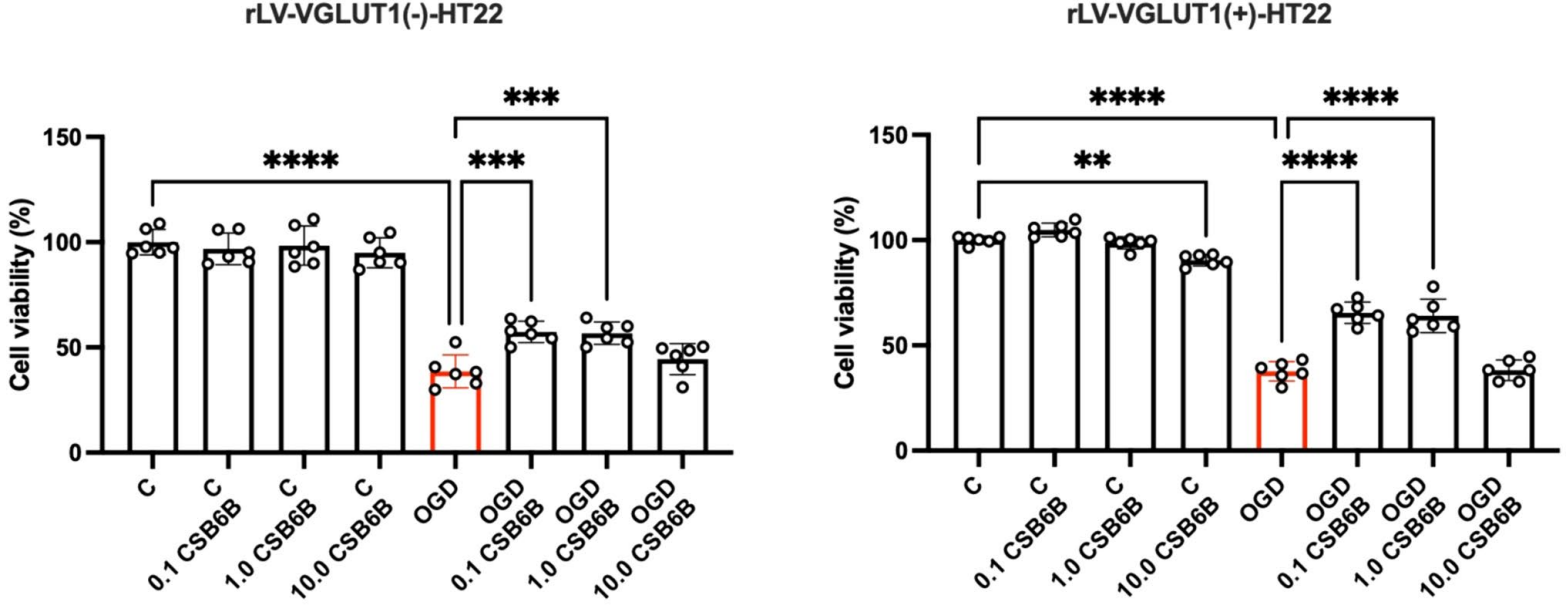
The effect of OGD and CSB6B treatment on cell viability assayed using PrestoBlue test. On the left results obtained for cell line transduced with blank construct (rLV-VGLUT1(-)-HT22), on the right results for cells with VGLUT1 overexpression (rLV-VGLUT1( +)-HT22). All data are presented as the mean ± SD. * p < 0.05 vs OGD, **** p < 0.0001 vs C or OGD, (one-way ANOVA, Sidak’s post hoc test, n = 7). *C* control group, *OGD* oxygen glucose deprivation, *CSB6B* Chicago Sky Blue 6B

Following OGD, a significant decrease in cell viability was observed (p < 0.0001 vs. control cells) (Fig. 2), indicating the detrimental impact of OGD on HT22 cells. In control cells not subjected to OGD, the highest concentration of CSB6B (10 μM) also decreased cell viability (p < 0.01 vs. control cells). However, in cells subjected to OGD, CSB6B at concentrations of 0.1 μM and 1.0 μM, but not 10 μM, significantly improved cell viability (p < 0.0001 vs. OGD). Interestingly, in cells transduced with rLV containing an empty vector, CSB6B at the same concentrations also showed a protective effect on cells after OGD.

### The Effect of VGLUT1 Inhibition on LDH Levels in Combined Culture Medium After OGD and Reoxygenation was Investigated

**Fig. 3:**
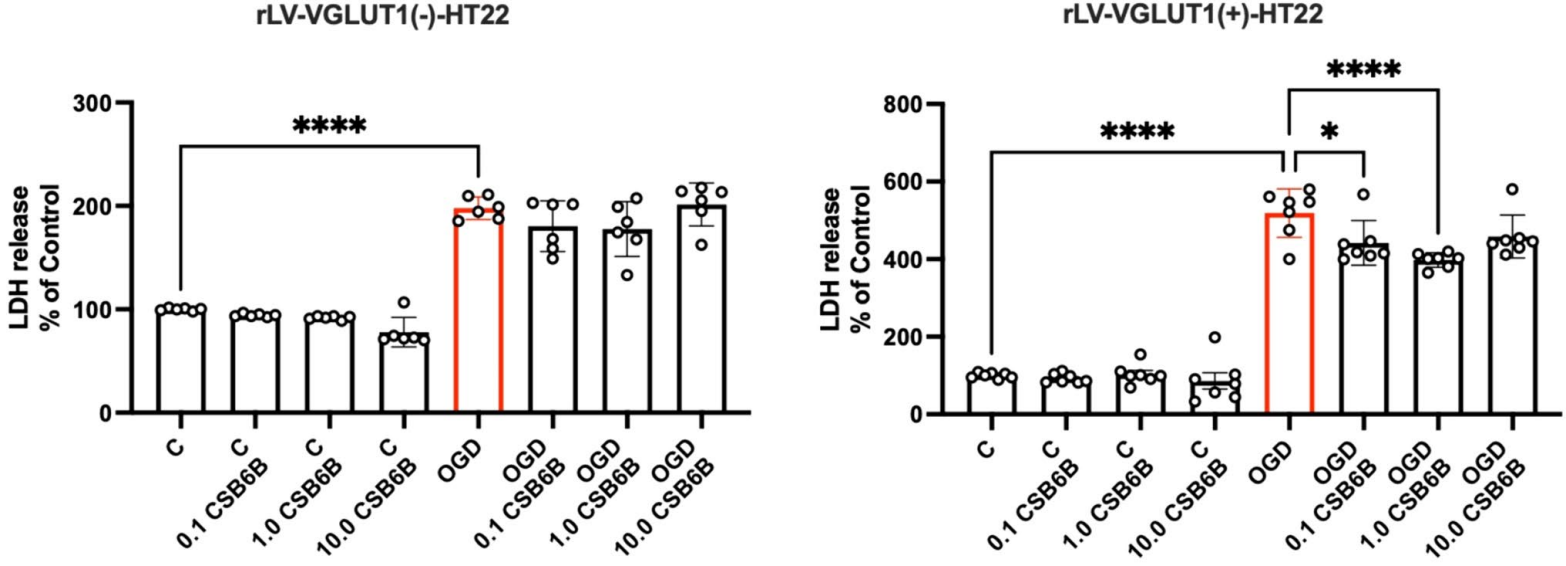
The effect of OGD and CSB6B on the level of LDH release. On the left results obtained for cell line transduced with blank construct (rLV-VGLUT1(-)-HT22), on the right results for cells with VGLUT1 overexpression (rLV-VGLUT1( +)-HT22). All data are presented as the mean ± SD. *p < 0.05 vs OGD, ****p < 0.0001 vs C or OGD, (one-way ANOVA, Sidak’s post hoc test, n = 6–7). *C* control group, *OGD* oxygen glucose deprivation, *CSB6B* Chicago Sky Blue

In cells exposed to OGD, LDH release was significantly elevated (p < 0.0001 vs. control) (Fig. 3). CSB6B did not affect LDH concentration in control cells not subjected to OGD. However, in cells exposed to OGD and CSB6B at concentrations of 0.1 μM and 1 μM, a reduction in LDH release was observed (p < 0.05 and p < 0.001, respectively, vs. OGD). In cells transduced with rLV containing an empty vector, CSB6B at the same concentrations did not show any protective effect on control cells or cells subjected to the OGD procedure. Noteworthy, in cells transduced with empty vector, OGD elevated LDH level to the much lesser extend when comparing to cells with overexpression of VGLUT1 subjected to OGD.

### The Effect of CSB6B on Mitochondrial Membrane Potential was Assayed Using the JC-1 Method

**Fig. 4:**
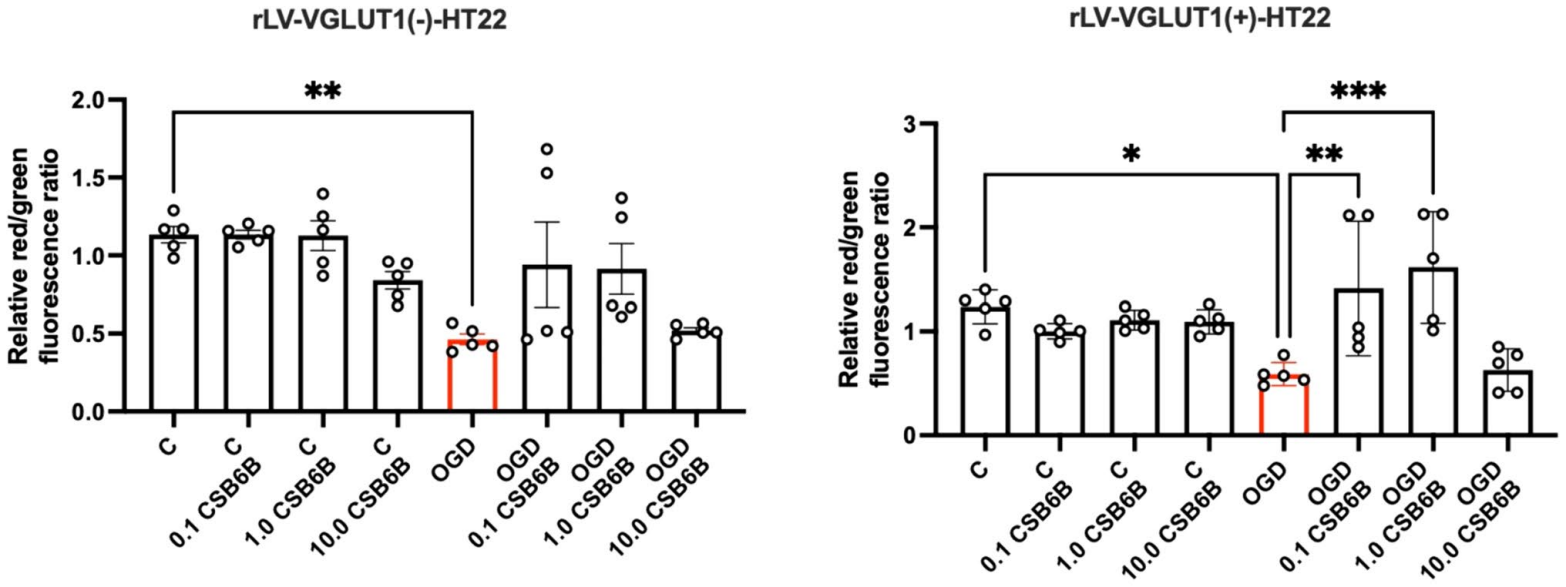
The effect of OGD and CSB6B on the mitochondrial membrane potential assayed with JC-1 test. On the left results obtained for cell line transduced with blank construct (rLV-VGLUT1(-)-HT22), on the right results for cells with VGLUT1 overexpression (rLV-VGLUT1( +)-HT22). All data are presented as the mean ± SD. *p < 0.05 vs OGD, **p < 0.01, ***p < 0.001 vs C or OGD, (one-way ANOVA, Sidak’s post hoc test, n = 5). *C* control group, *OGD* oxygen glucose deprivation, *CSB6B* Chicago Sky Blue 6B

**Fig. 5:**
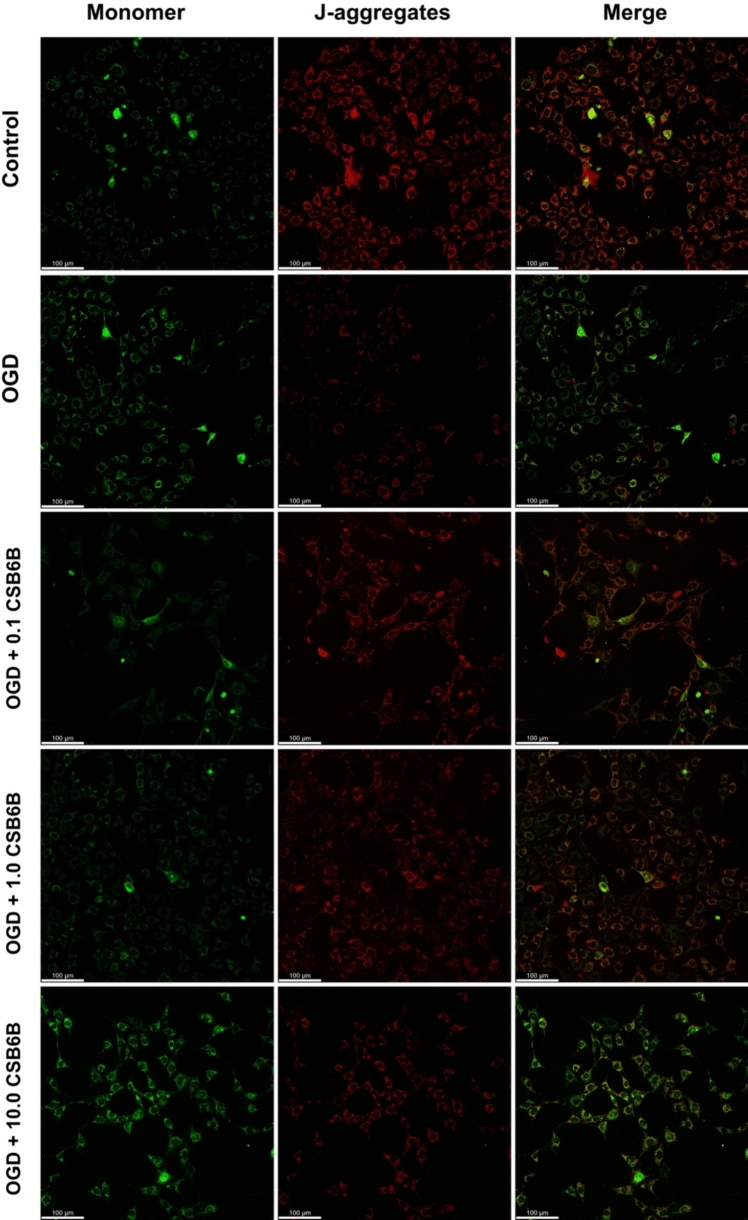
The effect of OGD and CSB6B on the mitochondrial membrane potential of HT22 cells with VGLUT1 overexpression, assayed with JC-1 test and visualized with confocal microscopy. *C* control group, *OGD* oxygen glucose deprivation, *CSB6B* Chicago Sky Blue 6B. The scale bar = 50 μm

In cells exposed to OGD, a decrease in mitochondrial membrane potential was observed (p < 0.05 vs. control) (Fig. 4). Cells exposed to OGD and treated with CSB6B at concentrations of 0.1 μM or 1.0 μM were significantly secured from the reduction of the mitochondrial membrane potential (p < 0.01 and p < 0.001, respectively, vs. OGD). These results were also visualized using confocal imaging (Fig. 5). In cells transduced with the blank vector, OGD also resulted in a reduction of mitochondrial membrane potential (p < 0.01 vs. control), but no significant effect of CSB6B treatment was observed.

### The Effect of CSB6B on the Expression Levels of Proteins Associated with Hypoxia-Induced Damage—AIF, NLRP3, and PARP1—was Evaluated in Cells Overexpressing VGLUT1

**Fig. 6:**
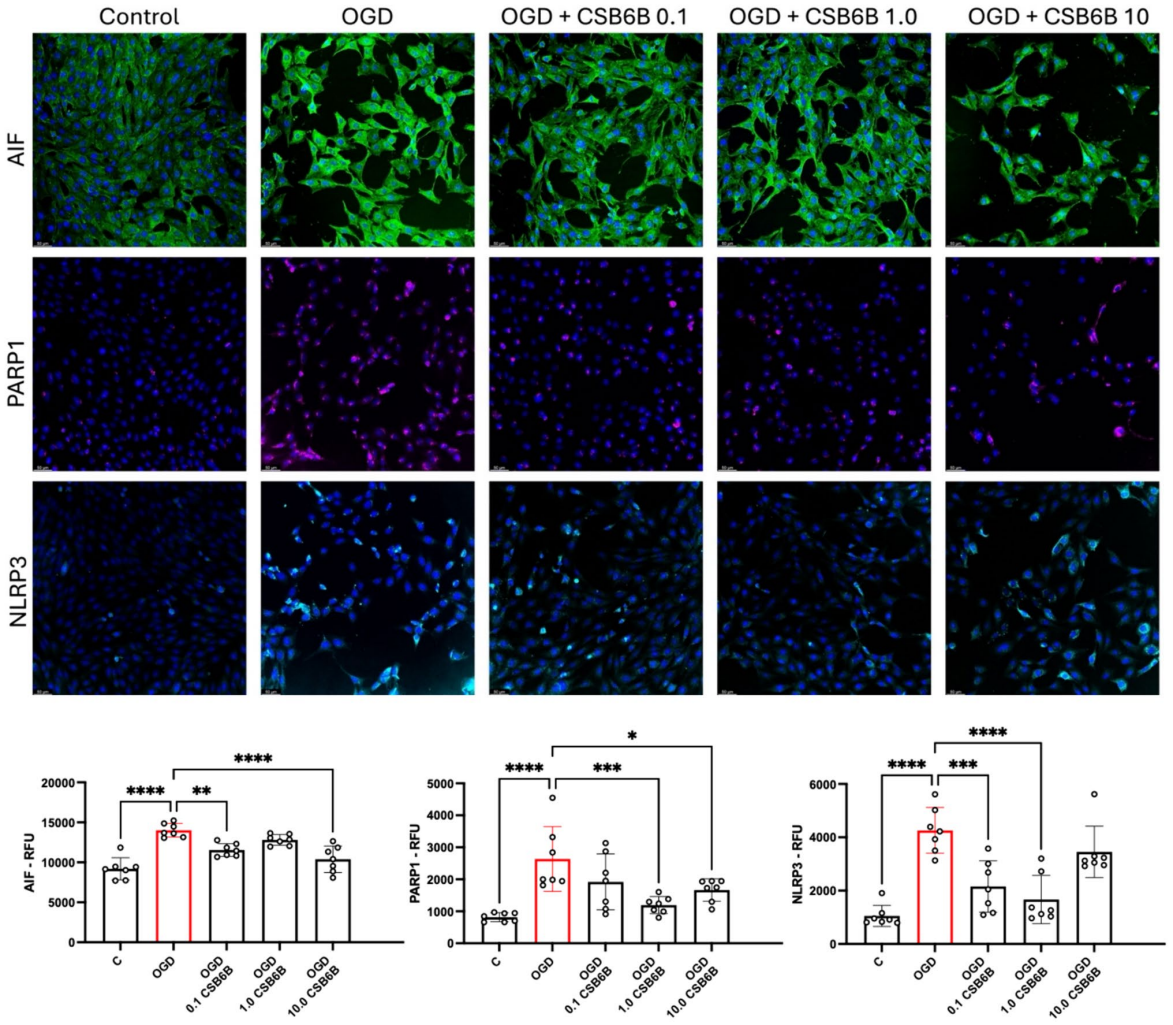
The effect of OGD and the treatment with CSB6B, on the level of AIF, PARP1 and NLRP3 proteins in HT22 cells overexpressing VGLUT1. All data are presented as the mean ± SD. *p < 0.05 vs OGD, **p < 0.01, ***p < 0.001, ****p < 0.0001 vs C or OGD, (one-way ANOVA, Sidak’s post hoc test, n = 7). *C* control group, *OGD* oxygen glucose deprivation, *CSB6B* Chicago Sky Blue 6B, *RFU* relative fluorescence unit. The scale bar = 50 μm

Under OGD conditions, HT22 cells with VGLUT1 overexpression exhibited significantly elevated expression of AIF, NLRP3, and PARP1 (p < 0.0001 vs. control) (Fig. 6). Treatment with CSB6B at concentrations of 0.1 and 10 μM resulted in reduced levels of AIF following OGD and reoxygenation (p < 0.01 and p < 0.0001 vs. OGD, respectively). The effect on cleaved PARP1 was observed at concentrations of 1.0 and 10.0 μM, with treatment reducing the levels of this protein (p < 0.001 and p < 0.05 vs. OGD, respectively). Additionally, CSB6B treatment at concentrations of 0.1 and 1.0 μM significantly reduced the levels of NLRP3 (p < 0.001 and p < 0.0001 vs. OGD, respectively) (Fig. 6).

## Discussion

VGLUTs play a crucial role in the CNS by regulating the Glu transport into synaptic vesicles, which is essential for synaptic transmission (Krzyzanowska et al., 2014). Their function is particularly important in the context of many neurological diseases, including stroke. In stroke, VGLUTs are involved in the glutamatergic neurotransmission that can lead to excitotoxicity, a condition where excessive Glu causes neuronal damage and death. VGLUT1 and VGLUT2 have been shown to influence the extent of Glu release and subsequent excitotoxic damage (Takamori et al., 2000). Therefore, targeting VGLUTs activity could provide therapeutic benefits in mitigating the effects of stroke and other neurological diseases (Pomierny et al., 2023; Yan et al., 2020). In our previous studies we showed in in vivo model of brain ischemia, that unspecific inhibition of VGLUTs, reduced neurological deficit, infarct volume as well as reduced excitotoxicity and neuroinflammation in rats (Pomierny et al., 2023, 2024). Unfortunately, due to high homology of VGLUTs, there are no specific and selective inhibitors of VGLUT1, which is known as a main vesicular transporter in the CNS (Thompson et al., 2005). In this study, we used CSB6B as an unselective VGLUTs inhibitor in an in vitro model of ischemia/reperfusion injury—the OGD model. We used HT22, immortalized hippocampal neurons with rLV the overexpression of VGLUT1, thus limiting the inhibitory effect of CSB6B “nearly selectively” to this transporter. The wild-type HT22 cells showed the native expression of VGLUT1 (Fig. 1) however, at barely detectable level.

In our study, we demonstrated that cells overexpressing the VGLUT1 protein released more than twice the amount of LDH in response to OGD (Fig. 3). Since LDH is a marker of cytotoxicity, this suggests that elevated VGLUT1 expression in HT22 cells during OGD may be a crucial excitotoxic trigger for cell degeneration. Notably, inhibiting transporters with 0.1 μM and 1.0 μM of CSB6B in HT22 cells overexpressing VGLUT1 completely prevented LDH release, an effect not observed in cells with native VGLUT1 expression. Interestingly, CSB6B maintained cell viability in both cell cultures subjected to OGD, regardless of VGLUT1 overexpression. This phenomenon is challenging to explain with the current data, especially since CSB6B did not affect cells with native VGLUT1 expression under OGD conditions in other tests. The neuroprotective effect of VGLUT1 blockage is well pronounced in test measuring the mitochondrial membrane potential. In this test we verified results of quantitative fluorescence readout by visualizing analyzed cells with confocal microscopy. Both cell cultures in OGD conditions showed significantly reduced potential of mitochondrial membrane. However, only in cells overexpressing VGLUT1, CSB6B (0.1 μM and 1.0 μM but not 10 μM) secured mitochondrial potential at basal level. In Glu-mediated excitotoxicity, degradation of mitochondria in postsynaptic cells is one of the key factors determining the fate of the neuronal cell in ischemic conditions (Ye et al., 2013). Mitochondrial dysfunction also initiates oxidative stress, release of Ca^2+^, caspases, but also release of AIF, and activation of NLRP3 (Feng et al., 2020). Elevated levels of reactive oxygen species (ROS) and activated AIF contribute to chromatin condensation and DNA damage, subsequently activating poly(ADP-ribose) polymerase 1 (PARP1), an enzyme involved in DNA repair (Liu et al., 2022). However, in terms of limited energy supply during OGD, overactivated PARP1 may even accelerate in time the energy deficit, what may eventually end up with necrosis. Indeed, in this study OGD conditions raised the level of cleaved PARP1, AIF and NLRP3. The treatment with CSB6B at studied concentrations resulted in reduced level of these proteins in response to OGD conditions, what may suggest the neuroprotective effect of VGLUT1 inhibition.

This study demonstrated that inhibiting VGLUT1 may have neuroprotective benefits. However, several limitations need to be addressed in future research. First, other isoforms of VGLUTs, such as VGLUT2 and VGLUT3, should be studied in the similar way. It is also necessary to correlate the effects of CSB6B treatment on VGLUT1 activity and resulting neuroprotection. These extended studies, along with our current and previous findings, could provide a comprehensive understanding of the role of VGLUTs in brain ischemia and potentially lead to the development of innovative treatments for neurological disorders like stroke.

## Data Availability

Data will be made available on reasonable request.

## References

[CR1] Choi, D. W. (2020). Excitotoxicity: Still hammering the ischemic brain in 2020. *Frontiers in Neuroscience,**14*, 579953. 10.3389/FNINS.2020.579953/BIBTEX 33192266 10.3389/fnins.2020.579953 PMC7649323

[CR2] Dong, X. X., Wang, Y., & Qin, Z. H. (2009). Molecular mechanisms of excitotoxicity and their relevance to pathogenesis of neurodegenerative diseases. *Acta Pharmacologica Sinica,**30*(4), 379. 10.1038/APS.2009.24 19343058 10.1038/aps.2009.24 PMC4002277

[CR3] Feng, Y. S., Tan, Z. X., Wang, M. M., Xing, Y., Dong, F., & Zhang, F. (2020). Inhibition of NLRP3 inflammasome: A prospective target for the treatment of ischemic stroke. *Frontiers in Cellular Neuroscience,**14*, 526042. 10.3389/FNCEL.2020.00155/BIBTEX 10.3389/fncel.2020.00155 PMC7283578 32581721

[CR4] Fremeau, R. T., Troyer, M. D., Pahner, I., Nygaard, G. O., Tran, C. H., Reimer, R. J., Bellocchio, E. E., Fortin, D., Storm-Mathisen, J., & Edwards, R. H. (2001). The expression of vesicular glutamate transporters defines two classes of excitatory synapse. *Neuron,**31*(2), 247–260. 10.1016/S0896-6273(01)00344-0 11502256 10.1016/s0896-6273(01)00344-0

[CR5] Kaneko, T., & Fujiyama, F. (2002). Complementary distribution of vesicular glutamate transporters in the central nervous system. *Neuroscience Research,**42*(4), 243–250. 10.1016/S0168-0102(02)00009-3 11985876 10.1016/s0168-0102(02)00009-3

[CR6] Krzyzanowska, W., Pomierny, B., Filip, M., & Pera, J. (2014). Glutamate transporters in brain ischemia: To modulate or not? *Acta Pharmacologica Sinica,**35*(4), 444–462. 10.1038/aps.2014.1 24681894 10.1038/aps.2014.1 PMC4813728

[CR7] Liu, L., Li, J., Ke, Y., Zeng, X., Gao, J., Ba, X., & Wang, R. (2022). The key players of parthanatos: Opportunities for targeting multiple levels in the therapy of parthanatos-based pathogenesis. *Cellular and Molecular Life Sciences,**79*(1), 1–15. 10.1007/S00018-021-04109-W 10.1007/s00018-021-04109-w PMC11073082 35000037

[CR8] Pellegrini-Giampietro, D. E., Meli, E., & Moroni, F. (2004). Excitotoxicity in cerebral ischemia. *Excitotoxicity in Neurological Diseases*. 10.1007/978-1-4419-8959-8_9

[CR9] Pomierny, B., Krzyżanowska, W., Skórkowska, A., Jurczyk, J., Budziszewska, B., & Pera, J. (2024). Chicago Sky Blue 6B exerts neuroprotective and anti-inflammatory effects on focal cerebral ischemia. *Biomedicine & Pharmacotherapy*. 10.1016/J.BIOPHA.2023.116102 10.1016/j.biopha.2023.116102 38159376

[CR10] Pomierny, B., Krzyżanowska, W., Skórkowska, A., Jurczyk, J., Bystrowska, B., Budziszewska, B., & Pera, J. (2023). Inhibition of vesicular glutamate transporters (VGLUTs) with Chicago Sky Blue 6B before focal cerebral ischemia offers neuroprotection. *Molecular Neurobiology,**60*(6), 3130–3146. 10.1007/s12035-023-03259-1 36802054 10.1007/s12035-023-03259-1 PMC10122628

[CR11] Shen, Z., Xiang, M., Chen, C., Ding, F., Wang, Y., Shang, C., Xin, L., Zhang, Y., & Cui, X. (2022). Glutamate excitotoxicity: Potential therapeutic target for ischemic stroke. *Biomedicine & Pharmacotherapy,**151*, 113125. 10.1016/J.BIOPHA.2022.113125 35609367 10.1016/j.biopha.2022.113125

[CR12] Takamori, S., Rhec, J. S., Rosenmund, C., & Jahn, R. (2000). Identification of a vesicular glutamate transporter that defines a glutamatergic phenotype in neurons. *Nature,**407*(6801), 189–194. 10.1038/35025070 11001057 10.1038/35025070

[CR13] Thompson, C., Davis, E., Carrigan, C., Cox, H., Bridges, R., & Gerdes, J. (2005). Inhibitors of the glutamate vesicular transporter (VGLUT). *Current Medicinal Chemistry,**12*(18), 2041–2056. 10.2174/0929867054637635 16101493 10.2174/0929867054637635

[CR14] Yan, S., Xuan, Z., Yang, M., Wang, C., Tao, T., Wang, Q., & Cui, W. (2020). CSB6B prevents β-amyloid-associated neuroinflammation and cognitive impairments via inhibiting NF-κB and NLRP3 in microglia cells. *International Immunopharmacology*. 10.1016/j.intimp.2020.106263 32028243 10.1016/j.intimp.2020.106263

[CR15] Ye, H. B., Shi, H. B., & Yin, S. K. (2013). Mechanisms underlying taurine protection against glutamate-induced neurotoxicity. *Canadian Journal of Neurological Sciences,**40*(5), 628–634. 10.1017/S0317167100014840 10.1017/s0317167100014840 23968934

